# Ultraconformable cuff implants for long-term bidirectional interfacing of peripheral nerves at sub-nerve resolutions

**DOI:** 10.1038/s41467-024-51988-1

**Published:** 2024-08-30

**Authors:** Alejandro Carnicer-Lombarte, Alexander J. Boys, Amparo Güemes, Johannes Gurke, Santiago Velasco-Bosom, Sam Hilton, Damiano G. Barone, George G. Malliaras

**Affiliations:** 1 University of Cambridge, Electrical Engineering Division, 9 JJ Thomson Ave, Cambridge, CB3 0FA United Kingdom; 2 University of Cambridge, Department of Chemical Engineering and Biotechnology, Cambridge, CB2 0QQ United Kingdom; 3 University of Potsdam, Institute of Chemistry, Karl-Liebknecht-Str. 24-25, 14476 Potsdam, Germany; 4 University of Cambridge, School of Clinical Medicine, Department of Clinical Neurosciences, Cambridge Biomedical Campus, Cambridge, CB2 0QQ United Kingdom

**Keywords:** Biomedical engineering, Brain-machine interface

## Abstract

Implantable devices interfacing with peripheral nerves exhibit limited longevity and resolution. Poor nerve-electrode interface quality, invasive surgical placement and development of foreign body reaction combine to limit research and clinical application of these devices. Here, we develop cuff implants with a conformable design that achieve high-quality and stable interfacing with nerves in chronic implantation scenarios. When implanted in sensorimotor nerves of the arm in awake rats for 21 days, the devices record nerve action potentials with fascicle-specific resolution and extract from these the conduction velocity and direction of propagation. The cuffs exhibit high biocompatibility, producing lower levels of fibrotic scarring than clinically equivalent PDMS silicone cuffs. In addition to recording nerve activity, the devices are able to modulate nerve activity at sub-nerve resolution to produce a wide range of paw movements. When used in a partial nerve ligation rodent model, the cuffs identify and characterise changes in nerve C fibre activity associated with the development of neuropathic pain in freely-moving animals. The developed implantable devices represent a platform enabling new forms of fine nerve signal sensing and modulation, with applications in physiology research and closed-loop therapeutics.

## Abstract (web summary)

Nerve interfaces have the potential to deliver therapythroughout the body by monitoring and modulating function via the peripheral nervous system. Here, the authors present an ultraconformable cuff capable of high-resolution recording and stimulation of nerve activity in freely-moving conditions.

## Introduction

The peripheral nervous system (PNS) is formed by a vast signal-transmitting network linking the central nervous system (CNS) with most organs and structures in the body. Nerves provide the CNS with information on the state of the structure to which they connect and transmit modulation signals back to the periphery. This wide anatomical distribution and unique physiological role make nerves an attractive target for interfacing with implantable devices^1–3^. Nerve implants can be deployed to a particular nerve to gain information on the physiological state of the organ it innervates via electrical recording, or to modulate the function of that organ through electrical stimulation. This flexibility has allowed their use in a multitude of applications^4–7^.

While the research and treatment opportunities offered by nerve interfacing are substantial, translation into applications has remained limited due to the inadequate long-term performance of most interfaces^8^. Implantable nerve interfaces are generally classified based on their location in or around nerves. Low-invasive epineural interfaces – usually in the form of cuffs – wrap around the circumference of the nerve and interface using one or few electrodes providing low selectivity recording and stimulation capabilities^1,8^. Cuffs are used in the clinic for whole-nerve stimulation therapies such as vagus nerve stimulation^6,7^. However, most nerves are composed of fascicles connecting to multiple targets, making whole-nerve interfacing unsuitable for many recording and stimulation applications. As an alternative to cuffs, intraneural or penetrating nerve implants pierce the nerve epineurium and deploy electrodes throughout the endoneurium^1^. Their more invasive design allows for the recording and stimulation of select portions of the nerve, enabling a wider range of applications. However, the higher tissue damage caused by penetrating devices and their reliance on a close interface with axons makes them highly vulnerable to chronic inflammation and foreign body reaction (FBR) – a slow-developing process affecting both cuffs and penetrating implants leading to their envelopment in a fibrotic scar^8^. FBR and tissue-implant interface degradation limit the long-term use of penetrating nerve interfaces, particularly for recording applications^2,8^.

Advances in materials and microfabrication strategies have opened new opportunities to interface with the nervous system. The last decade has seen the development of a wide range of multielectrode array implants interfacing with nervous tissue in novel ways^9^, with a growing focus on soft and flexible constructions to improve tissue interface quality and long-term stability^9–14^, as well as high-performance recording microelectrode materials^11^. However, most of these technologies have focused on the CNS. Peripheral nerve interface designs have benefitted comparatively less from advances in fabrication. While penetrating nerve interfaces with flexible materials and microelectrode arrays have been developed^15,16^, nerve cuffs in particular have seen little innovation, with many applications continuing to rely on few large electrodes and rigid thick-film-fabricated materials^7^. This has limited their use in long-term applications despite their more clinically-translatable lower-invasive design.

In this work we present an implantable nerve cuff with a conformable design which exhibits long-term tissue compatibility and stable recording of nerve activity for longer periods of time. We show that these cuffs can achieve sub-nerve recording and stimulating resolutions typically limited to more invasive penetrating implants, as well as the ability to identify axon conduction velocity and direction in awake animals. We finally demonstrate the potential of this technology as a research and therapeutic tool by employing it to identify and characterise over time changes in nociceptive C fibre activation in a partial nerve ligation model of neuropathic pain.

## Results

### Design and fabrication of conformable cuffs

**Fig. 1:**
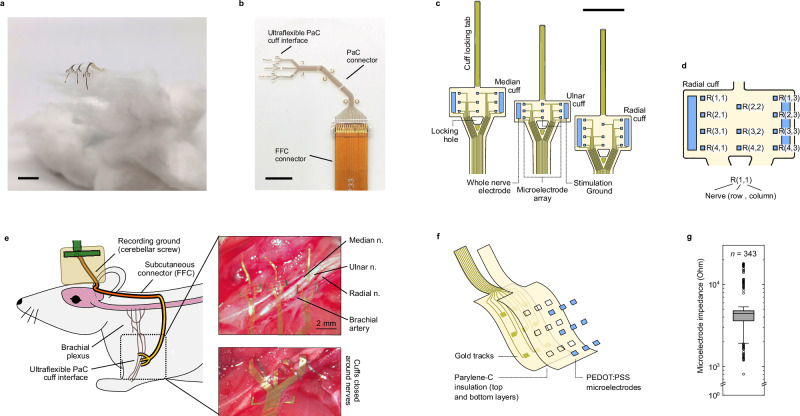
Overview of ultraconformable nerve cuff arrays. **a** Photograph of nerve cuff array device on a cotton ball, highlighting its flexible and lightweight construction. **b** Photograph of nerve cuff array device. The cuffs are fabricated from parylene C (PaC). These carry electrode connections through a PaC connector, bonded to a flat flexible cable (FFC) connector through which they are externalised. The shape of the PaC connector is designed to accommodate the shape and movement of the rat shoulder, and contains multiple tabs to facilitate surgical manipulation and suturing. **c** Diagram of cuff and multielectrode array layout. Each implantable device consists of three cuffs designed for implantation into three individual nerves (median, ulnar, and radial nerves). Each cuff consists of a regular array of 100 by 100 μm microelectrodes which are wrapped around the circumference of the nerve during implantation. To seal each cuff, its locking tab is fed through the cuff’s locking hole. In addition to the microelectrodes, each cuff contains a larger electrode which can be used for whole nerve stimulation/recording and a ground used for nerve stimulation. **d** Diagram displaying naming convention of microelectrodes (radial nerve cuff shown). Note the radial nerve cuff lacks a R(1,2) microelectrode due to device channel number limitations. Microelectrodes within the same column in the array are deployed as a ring around the cuffed nerve when implanted, and are referred to as rings instead of columns in in vivo experiments. **e** Diagram of chronic implantation model and images of device implantation into nerves of the rat forearm. Implanted cuffs microelectrode connections are externalised via a subcutaneously-positioned FFC, exiting the body of the rat through a headcap port. For recording, a ground is implanted into the CSF above the rat cerebellum. **f** Construction of nerve cuff array device. The device consists of gold tracks encapsulated between two layers totalling to 4 μm of PaC. The tracks connect to PEDOT:PSS microelectrodes. **g** Impedance of device microelectrodes in saline following fabrication (*n* = 343 microelectrodes, *N* = 12 devices). Scale bar (**a**, **b**): 10 mm, c: 2 mm. Box represents interquartile range, inner mark represents median, whiskers represent 1.5 times the interquartile range, outliers beyond these are represented as circles. Source data for (**g**) are provided as a Source Data file.

We fabricated our implantable cuffs using thin (4 μm) parylene C (PaC) as a substrate. This rendered the devices highly flexible, allowing them to conform well to curved surfaces without damaging them (Fig. 1a). Wiring connections to the poly(3,4-ethylenedioxythiophene):poly(styrenesulfonate) (PEDOT:PSS) microelectrode array were carried by a narrow PaC connector. The shape of this connector was designed to accommodate the anatomy of the rat forelimb, and included suture loops and surgical guidance tabs to facilitate handling and securing the cuffs after implantation (Fig. 1b). Connections were then carried to a flat flexible cable (FFC) connector.

Our implants were designed with a triple cuff construction to interface with three key nerves of the brachial plexus: the median, ulnar and radial nerves (Fig. 1c). These three nerves are responsible for movement and sensation in the hand of both humans and rats^17,18^, and are being investigated as interfacing targets to restore hand function^15,19^. The three cuffs had a small profile with a width of 2.6 mm each, and lengths of 1.35, 1.0 and 1.6 mm tailored to the circumferences of the three nerves in rats. The cuffs were designed with long gold-reinforced tabs to facilitate implantation and cuff closure through a locking hole at the cuff base (Fig. 1c). Closed cuffs could be sealed by applying a drop of fast-curing silicone at the tip of these tabs, thereby maintaining the cuffs devoid of any sealing materials to provide enhanced conformability to the nerve after implantation. Each cuff contained an array of 100 by 100 μm PEDOT:PSS microelectrodes and two larger electrodes which enveloped the whole nerve – one used to record whole-nerve activity and one used as a ground to allow for local stimulation setups (Fig. 1c, d). Our choice of electrode dimensions and layout was similar to those of multielectrode cuffs able to record^20^ and stimulate at high resolutions^21^.

The fabricated designs could be easily implanted into the nerves at the upper portion of forelimbs of rats (Fig. 1e). The high flexibility and low profile of the device allowed cuffs to be implanted into the adjacent median, ulnar and radial nerves without the need for extensive surgical dissection, and could be conformed around shoulder muscles to facilitate normal limb movement post-implantation. Once the device reached the back of the animal, connections on PaC were replaced by the more robust FFC which was capable of supporting the larger displacements present in this part of the anatomy. This allowed the connections to be externalised through a headcap connection port.

The combination of a PaC substrate with gold connections and PEDOT:PSS microelectrodes has been shown to be robust for chronic recording in the more protected environment of the brain^22^. We adapted this microfabricated layer architecture (Fig. 1f) to accommodate the more challenging movement-rich environment of the peripheral anatomy and the forelimb in particular. The low impedance of PEDOT:PSS microelectrodes seen in other applications was also seen in our devices, with median impedance values of 5.47 ± 4.1 kOhm (mean ± standard deviation) when measured in saline (Fig. 1g), and 41.64 ± 128.06 kOhm when measured in an in vivo environment (implanted into nerves of the forearm in rat).

### Long-term tissue compatibility of cuff implants

**Fig. 2:**
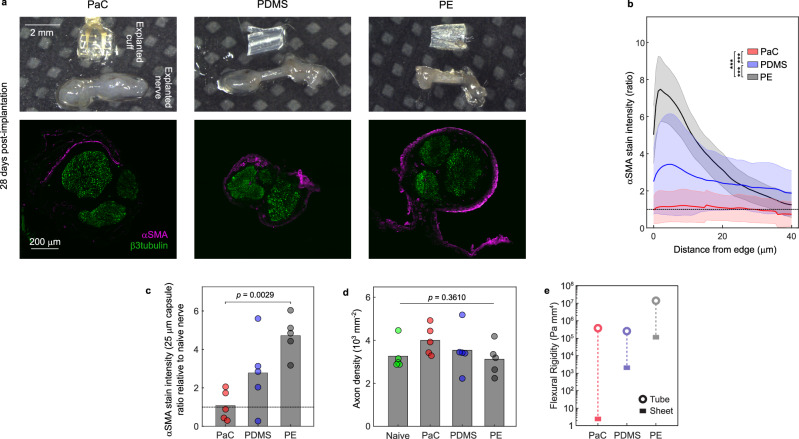
Ultraconformable PaC nerve cuffs exhibit minimal foreign body reaction following four weeks of implantation. **a** Photographs and immunohistological characterisation of nerve cross-section images of explanted cuffs and radial nerves four weeks after implantation. Nerves implanted with PaC cuffs with conformable design exhibit little fibrosis (αSMA) when compared to stiff polyethylene (PE) cuffs but also soft silicone (PDMS) cuffs. Axons stained by β3tubulin to show their position relative to the edge of the nerve. **b** Quantification of fibrosis (αSMA stain) at varying depths along the nerve circumference. Distance from edge represents 0 as tissue in direct contact with the cuff wall. Stain intensity presented as a ratio of stain relative to non-fibrosed tissue at the centre of the nerve. PaC cuffs cause lower fibrosis than both PE and PDMS cuffs and almost no fibrosis above background levels (deep nerve endoneurium). Lines represent group mean and shaded areas standard deviation. *N* = 5 rats. ***: *p* < 0.001, two-way ANOVA and Tukey post-hoc test. **c** Quantification of fibrosis (αSMA stain) at the 25 μm of nerve tissue closest to the cuffs. PaC cuffs cause a significantly lower degree of fibrosis than PE cuffs (*p* = 0.0029, ANOVA and two-tailed Tukey post-hoc test). *p* values for comparisons with *p* > 0.05 not included in graph. Bars represent the group mean and circles represent values for individual animals. **d** Quantification of axon density (β3tubulin stain) within the nerve cross-section of all three implant conditions compared to non-implanted naïve nerves. No significant difference in axon density is observed (*p* = 0.3610, one-way ANOVA). Bars represent the group mean and circles represent values for individual animals. *N* = 5 rats for (**c**) and (**d**). **e** Flexural rigidity for the different cuffs for a tube (open circle) and for a sheet (rectangle). Dashed line represents the expected range of flexural rigidity between these extremes. Flexural rigidity is calculated as the product of Young’s modulus and moment of inertia for the different shapes. The calculations are detailed in Supplementary Fig. 1. PaC exhibits a lower flexural rigidity range than both PDMS and PE due to its low thickness, providing the cuffs with high flexibility, conformability, and long-term tissue compatibility. Source data for (**b**–**e**) are provided as a Source Data file.

To determine the suitability of the developed highly conformable cuffs for long-term applications, we examined the tissue response to them over 28 days of implantation into the radial nerve of rats. We compared the degree of FBR between the nerve epinerium and the inner surface of the PaC cuffs, characterised by αSMA-positive myofibroblasts^23^, to that of 28 day-implanted medical-grade polydimethylsiloxane (PDMS) and polyethylene (PE) cuffs (Fig. 2a). Both of these materials are biocompatible, with PDMS considered the gold-standard for nerve cuff implants due to its low stiffness^7,8,10^. Nerves implanted with both PDMS and PE cuffs developed a ~ 25 μm thick fibrotic capsule, with PDMS capsules exhibiting high variability but overall significantly lower αSMA stain than PE ones (Fig. 2b, c). In contrast, PaC cuff-implanted nerves exhibited a consistently near-absent degree of fibrosis with capsule αSMA barely above background stain values (mean capsule-to-background ratio for PaC of 1.08. PDMS: 2.78-fold increase, PE: 4.72-fold increase) and significantly lower than the other cuffs tested (Fig. 2b, c). None of the tested devices had a significant impact on axon density as compared to naïve nerves (Fig. 2d, naïve: 3267 ± 675, PaC: 3998 ± 687, PDMS: 3536 ± 1057, PE: 3120 ± 742, axons per mm^2^, mean ± standard deviation).

To understand the observed variation in FBR, we examined the mechanics for each cuff type. Mechanical mismatch between implant and tissue is generally recognised as the major driver of FBR and tissue damage, particularly in the nervous system^8,10,24–26^. First, we determined the stiffness for each material (Young’s modulus PaC: 1.13 GPa, PDMS: 2.86 MPa, PE: 217 MPa, *n* = 4 − 7 samples) (Supplementary Fig. 1a–d), finding no trend with observed FBR. We used these values to calculate the flexural rigidity for each cuff, which also takes geometrical effects into account^27^. Given the wide range of loading conditions that could be present in the complex loading environment for a nerve cuff, we calculated a range of possible flexural rigidities that represent the different extremes (Supplementary Fig. 1e). On one extreme, we assumed the bending of the entire nerve cuff as a tube and on the other extreme, we assumed a rectangular cross-section, representing a point force on a singular small section of the cuff (Fig. 2e). As the nerve has an average stiffness of ~60 Pa^28^, which results in a flexural rigidity of 0.38 Pa mm^4^ (calculating the moment of inertia for a tube), the nerve mechanics should be negligible for any of the experimental nerve cuff materials. Given these calculations, this range shows that the PaC cuff minimises implant-tissue mechanical mismatch from a flexural rigidity standpoint, likely causing the near-absent FBR observed around the nerves for this case.

While the tested PE and PDMS cuffs were much thicker than PaC ones (PE: 0.25 mm, PDMS 0.28 mm), PE and PDMS cuff dimensions were similar to those of thick-film fabricated nerve cuffs which typically employ these materials. Clinically-used nerve-stimulating cuffs have thicknesses in the millimetre range^7^. Although PDMS has worse insulating properties and is more difficult to fabricate into a thin film device than PaC, recent advances in brain and spinal cord implantable electrode arrays have employed PDMS with thicknesses of 100 – 200 μm^29,30^. These lower thickness values would result in PDMS devices with lower mechanical mismatch to nerves, suggesting an alternative design to produce similarly long-term tissue-compatible cuffs.

### Long-term nerve recordings with ultraconformable cuffs

**Fig. 3:**
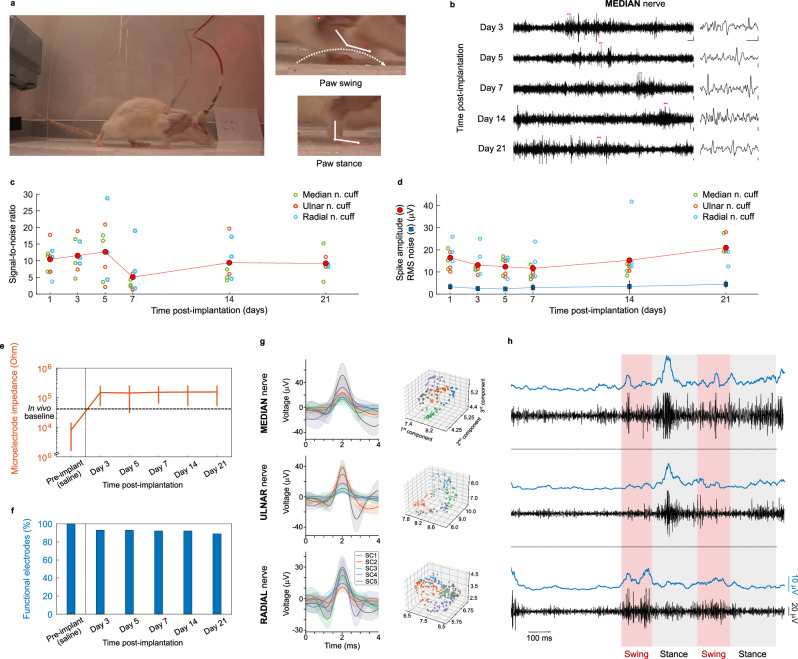
Ultraconformable nerve cuff arrays achieve stable recordings from their target nerves over chronic time periods. **a** Photographs of behaviour task performed by animals during electrophysiology recordings. Animals are recorded while walking down a transparent tunnel. This allows the identification of nerve activity related to the swing and stance phases of walking of the implanted limb. **b** Representative traces of recorded activity from the median cuff of a rat over a period of 21 days of implantation. Individual spikes can be seen throughout all timepoints (right, magnified from sections marked by red lines in traces on left). Scale bars: 100 ms and 10 μV (left) and 5 ms and 10 μV (right). **c**, **d** Scatter plots of signal-to-noise ratio (SNR) (**c**) and spike amplitude (**d**) of recorded traces from median (green open circles), ulnar (orange open circles) and radial (cyan open circles) cuffs, showing stable quality of recording over the 21 day implantation period. Average of all cuffs represented by filled red circles. Root mean squared (RMS) of noise shown in (**d**) (mean: blue squares, standard deviation: black lines, for all cuffs) to represent spike detection threshold. *n* = 9 cuffs (6 for day 21) from 3 rats (2 for day 21). One rat had to be culled past the 14 day timepoint due to skin damage caused by the FFC of the implant. **e**, **f** Microelectrode impedance (**e**) and survival (**f**) over the implantation period. Implantation leads to an impedance rise and the breakage of 7% of the microelectrodes, but these values remain thereafter stable. Device pre-implantation impedances are calculated in saline. Black dashed line indicates mean impedances of devices of the same format as those implanted measured in the in vivo environment, suggesting only a portion of the impedance increase seen at Day 3 is a consequence of changes in tissue-electrode interface. Functional microelectrodes are defined as having an impedance <1 MOhm. *n* = 96(/96 [100%] (Pre-implantation, interpreted as 96 out of 96 total microelectrodes, 100% functional microelectrodes), 89(/96) [92.7%] (Days 3–7), 88(/96) [91.7%] (Day 14), 56(/64) [87.5%] (Day 21, note one animal had to be culled before this timepoint for health issues not associated with the nerve cuff) microelectrodes, from 3 rats (2 for day 21). Line in (**e**) represents impedance mean, vertical bars represent impedance standard deviation. **g**, **h** Spike waveforms (**g**) and representative neural traces (black) and averaged RMS values (blue) (**h**) recorded from median, ulnar, and radial nerves simultaneously, 3 days post-implantation. Neural activity is seen to increase during the implanted limb stance phase of locomotion in median and ulnar nerves, and during the swing phase in radial nerve. Spike plots in (**g**) represent spike mean ± standard deviation. Source data for (**c**–**f**) are provided as a Source Data file.

To examine the performance of the developed implants for long-term in vivo use, we performed forearm nerve recordings over 21 days in awake rats. A cuff device implanted into the median, ulnar and radial nerves of the forelimb and with connections externalised through a headcap port was used to record nerve activity related to paw movement and sensation while the rat walked through a transparent tunnel (Fig. 3a). The cuffs were able to record action potential activity from nerves throughout the experiment period (Fig. 3b), with signal-to-noise ratios (SNR) and spike amplitudes remaining stable across the 21 days tested (Fig. 3c, d) (SNR across all timepoints: 9.7 ± 6.1, peak amplitude: 14.6 ± 6.6 μV, root mean squared noise: 3.1 ± 1.7 μV, mean ± standard deviation). Only small changes in microelectrode impedance were observed after chronic implantation, particularly when compared to values measured in in vivo environments (Fig. 3e), and little loss of functional electrodes was observed (Fig. 3f). Changes in impedance were not consistent with failure modes such as loss of PEDOT:PSS or Au delamination^31^, indicating robust long-term implant performance. Spike sorting identified activity from multiple sources within the recorded traces of each of the three interfaced nerve with different temporal evolutions over the behavioural task (Fig. 3g, Supplementary Fig. 2). Tight clustering was not observed in PCA plots of identified spikes. This may suggest that spikes were derived from a wide population of axons with activity corresponding to different sensory and motor functions. Nerve spike shapes and amplitudes were comparable to those observed in animals under anaesthesia^20,32^ and human microneurography recordings^33,34^.

Examination of the relation between the pattern of recorded activity with animal behaviour identified periods of high nerve activity during walking. More specifically, high activity in median and ulnar nerves was seen to coincide with the stance portion of walking locomotion, while radial nerve activity coincided with the swing phase (Fig. 3h). This pattern of activity matched the innervation pattern of the three nerves, with median and ulnar nerves modulating paw flexion movements and sensation from the palm^17,18^ – expected to predominantly occur during stance. Radial nerve, instead, predominantly mediates the paw extension expected during the swing phase of walk^18^. Moreover, the distinct difference in recorded activity pattern across electrodes on different adjacent nerves supports that recorded activity is neural in origin and not originating from other sources such as electromyogram – a common contaminant of peripheral nerve recording. Overall, microelectrodes recorded spikes at rates of 54.76 ± 9.61 spikes per second (*N* = 3 animals, mean ± standard deviation), although these varied depending on the activity of the animal.

The presence of the implants themselves caused no visible limitation in movement ability or dexterity of the paw. We observed a decrease in gripping ability over the first three days following implantation, likely associated with the surgical procedure, which recovered thereafter (Supplementary Fig. 3). Animals were overall able to move their paw and grip objects without issues with the presence of the implanted cuffs and cable (Supplementary Movie 1).

### Sub-nerve resolution recording and signal velocity sorting

**Fig. 4:**
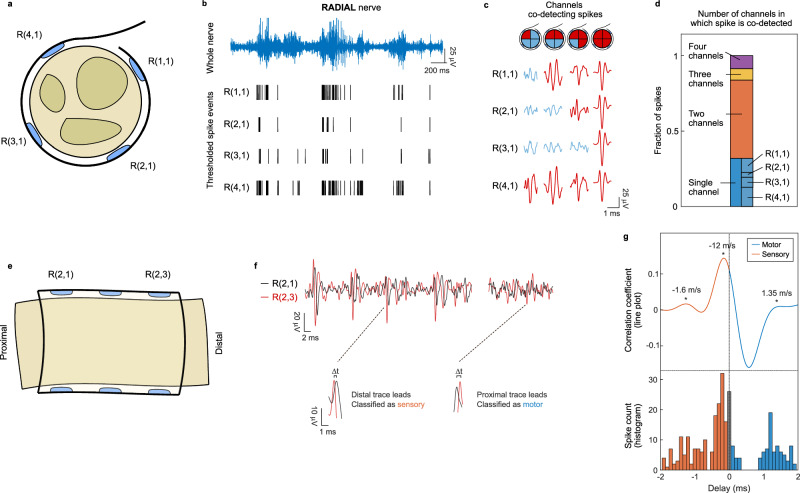
Ultraconformable cuff arrays achieve sub-nerve recording resolution without penetrating the nerve. **a** Diagram of layout of one ring of microelectrodes around the circumference of the radial nerve. **b** Spike events across four microelectrodes within a ring along the radial nerve circumference. The whole nerve recording is provided (blue) for reference. Spikes are detected in bursts in all microelectrodes, coinciding with the swing phase during rat walking. Some spikes are detected across all microelectrodes, while others are detected uniquely in some of them. **c** Sample recordings from four electrodes on a radial nerve microelectrode ring, demonstrating examples of spike co-detection across the microelectrode ring. Traces where a spike is identified are shown in red, while traces where no spike is detected are shown in blue. Diagrams at the top represent positions over the nerve cross-section where the spike is considered detected (red: detected, blue: not detected). **d** Quantification of spike coincidence across microelectrodes for activity shown in (**b**). Most neural spikes are detected across two microelectrodes simultaneously, or uniquely by a single microelectrode. All four microelectrodes record these unique spikes (breakdown of proportion represented in graph). Spikes are considered to be co-detected when they occur in several channels within 0.5 ms of each other. Co-detection is quantified by number of spikes, not by co-detection events (an event where four spikes are co-detected across all four microelectrodes is counted as four spikes). **e** Diagram of layout of microelectrodes positioned along the length of the radial nerve. **f** Neural activity recording from microelectrodes within two different rings in a cuff. Neural action potential spikes recorded across both rings exhibit a time delay, corresponding to their direction and velocity. This is highlighted at the bottom of the panel. **g** Quantifications of radial nerve activity delay between microelectrodes within the most proximal and most distal rings in the array of the cuff over a 2.7 s awake recording. Top: quantification corresponding to the cross-correlation value between the nerve recordings of the two microelectrodes at varying time delays. Bottom: histogram corresponding to the inter-spike interval between microelectrodes. Both analyses identify peaks of activity at delays corresponding to ~1.6 m/s afferent, ~12 m/s afferent, and ~1.35 m/s efferent, calculated based on the 2 mm distance between microelectrode rings. Fast afferent/efferent activity is also detected at close to 0 ms delay.

Implantable nerve cuffs are typically designed to record averaged activity from the whole nerve^1,2,8^. By using low-impedance sub-mm microelectrode arrays that conform optimally to the nerve surface, we hypothesised that our devices would be able to record unique neural activity from portions of the nerve. In order to test this, we compared the recordings of microelectrodes deployed as a ring around the same nerve (Fig. 4a).

In awake animals, all microelectrodes within the same ring recorded neural spikes in bursts which were synchronous across all microelectrodes (Fig. 4b, Supplementary Figs. 4, 5). However, analysis of these spike bursts revealed that bursts were made up of different neural spikes across different microelectrodes. These spikes could be recorded from anywhere between single to all four microelectrodes within the ring, with a co-detection pattern allowing for their location in space within the nerve cross-section (Fig. 4c). Coincidence analysis determined that most neural spikes were uniquely recorded by one or two microelectrodes within the ring, with few spikes being recorded across the entire ring of microelectrodes in a cuff (Fig. 4d, Supplementary Fig. 5). Moreover, all microelectrodes within a cuff ring could record unique spikes (Fig. 4d, Supplementary Fig. 5), indicating that spikes uniquely recorded by microelectrodes were not a result of poor performance of other microelectrodes in the ring. The median, ulnar, and radial nerves contain two or three fascicles each, and each connects to synergist muscles and adjacent skin dermatomes^18,35^. This likely gives rise to the spike pattern observed - microelectrodes within the same ring record bursts of activity corresponding to the same overall movement or sensation, with each microelectrode recording neural spikes from different axons and fascicles. Our results therefore show that the developed cuffs are able to record selectively from portions of the nerve without the need to pierce the epineurium.

Having confirmed sub-nerve recording resolution in microelectrodes deployed around the circumference of the nerve, we examined recordings along the length of the nerve. We made use of the precise microelectrode arrangement enabled by microfabrication and the high recording quality shown by our cuffs to carry out velocity sorting of nerve signals in awake animals. Microelectrodes positioned at different locations along the nerve epineurium recorded the same spikes but with some amount of delay (Fig. 4e, f). We interpreted this delay as arising from the specific conduction velocity of the axon giving rise to that action potential. We observed both spikes which lagged in the microelectrode positioned distally along the nerve – efferent nerve signals – as well as spikes which lagged in the proximal microelectrode – afferent nerve signals (Fig. 4f). The abundance of spiking activity seen in awake nerve recordings and compact design of the cuff microelectrode arrangement made velocity analysis techniques previously developed and tested under anaesthesia^36–40^ unsuitable for our awake recordings. We developed two analysis strategies to determine conduction velocity, the first involving spike detection through thresholding via an adapted inter-spike histogram, and the second involving a cross-correlation calculation between the two recorded traces which utilised the entire recorded waveform. Both of these analysis strategies yielded agreeing results (Fig. 4g, Supplementary Fig. 6). We identified several populations of signal velocities corresponding to known sensorimotor nerve fibre types^41^, slow sensory 10 - 30 m/s (Aδ fibre velocity), slowest sensory 1 - 3 m/s (C fibre velocity) and slow motor 1 - 3 m/s (Aγ fibre velocity). The limited sampling rate used in typical electrophysiology recording hardware and the compact design required in chronically implanted devices limited sorting capabilities for fast sensorimotor fibres > 30 m/s (Aβ and Aα fibre velocities). However, we identified populations of spikes with almost no delay between microelectrodes, which likely corresponded to these fast fibres. Our results validated the neural velocity sorting capabilities in awake, physiological environments for the developed highly conformable cuffs.

### Forelimb paw movement control using sub-nerve resolution stimulation

Implantable nerve interfaces are commonly utilised for neuromodulation applications through electrical stimulation. Similar to recordings, cuff interfaces are typically designed to stimulate whole nerves, while the more invasive penetrating interfaces selectively stimulate portions of the nerve^1,2,8^. We tested whether the developed cuffs could achieve selective stimulation of portions of the nerve, mirroring their recording capabilities, and comparable to the resolution achieved by penetrating devices.

**Fig. 5:**
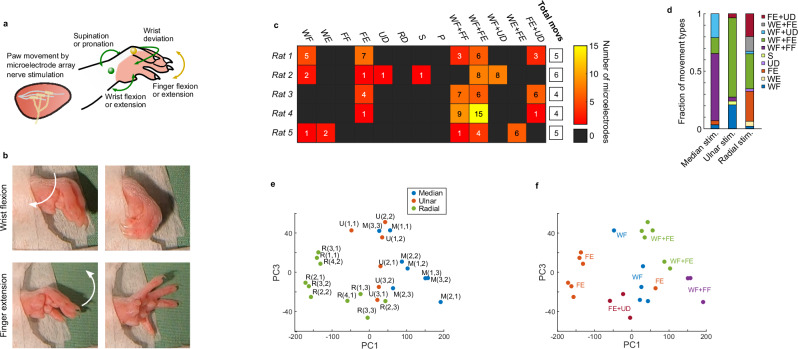
Ultraconformable cuff arrays achieve sub-nerve stimulation resolution. **a** Diagram of the experimental setup. Stimulation of the three implanted nerves using individual microelectrodes causes movements of the wrist and fingers of the paw, in rats under anaesthesia. **b** Pictures of examples of movements produced before (left) and during (right) nerve stimulation. **c** Heatmap of quantification of movements produced by microelectrode stimulation across five rats. While stimulation on occasion caused simple movements, more often combination movements (containing “+” in name) were observed. Certain simple movements were never observed. Despite variations in movements across different implantations and animals, more than three movements were observed in every rat, indicating higher than whole-nerve stimulation resolution. **d** Quantification of the types of movements produced by stimulation using microelectrodes on the three different nerves. **e**, **f** Plot of principal components of kinematic analysis of the various movements produced, for Rat 1. **e** Movements are tagged based on the microelectrode that produced them. Clustering is seen across groups of microelectrodes within each cuff. **f** Movements are tagged by the identified type of movement produced. Clustering indicates that many identified types of movements can be performed with different kinematics, indicating additional stimulation selectivity not captured in panel (**c**). WF: wrist flexion, WE: wrist extension, FE: finger extension, UD: ulnar deviation, SD: sacral deviation, S: supination, P: pronation, WF + FF: wrist flexion and finger flexion, WF + FE: wrist flexion and finger extension, WF + UD: wrist flexion and ulnar deviation, WE + FE: wrist extension and finger extension, FE + UD: finger extension and ulnar deviation, Total movs: total number of different types of movement produced.

We tested the neuromodulation capabilities of the cuffs by evaluating their ability to generate different forelimb paw movements (Fig. 5a, b). For this we employed models under anaesthesia, to prevent the confounding of implant-generated movements with those produced by the animals themselves. The median, ulnar and radial nerves are responsible for controlling hand movement, and nerve interfaces have been used in these nerves to restore hand function in amputation and spinal cord injury patients^15,19^. While rats have lower hand dexterity than primates, they are able to grasp objects with their forelimb paws. Testing in rats under anaesthesia showed that stimulation through the cuffs could achieve multiple different paw movements (Fig. 5c, Supplementary Fig. 7) even while using identical stimulation parameters throughout all electrodes (see Methods). While some variability in the range movements produced was observed across different implantations – likely due to small differences in nerve fascicle-microelectrode relative position – the cuffs could reliably produce movements including wrist and finger flexion as well as extension, which are key for hand function^15^. Although part of this movement selectivity was achieved through stimulation of the three different nerves, with flexion predominantly produced through median nerve stimulation and extension through radial nerve stimulation, stimulation of any one nerve using individual microelectrodes within the same cuffs could produce different movements (Fig. 5d). Overall, we observed more than three movements in all implantation experiments, with some implantations yielding up to six recognisable movements (Fig. 5c). These observations were indicative of the devices selectively stimulating portions of each nerve. We further confirmed this by performing an analysis of the movement kinematics, which showed that clusters of microelectrodes within the same nerve cuff could produce different movements (Fig. 5e, f, Supplementary Fig. 8).

As we could not explore activation characteristics of the forearm muscles via electromyogram recordings due to the small size of rats, we focused our analysis on the resulting hand movements. Our results confirmed that the developed implants were able of producing a variety of hand movements despite not penetrating into the nerve endoneurium. Moreover, while we only characterised movements produced by individual microelectrodes, we observed that these could be combined to produce more complex movements, either by simultaneous stimulation or activation in sequence (Supplementary Movie 2). The stimulation parameters such as pulse train duration could also be altered to produce faster, shorter-lasting movements (Supplementary Movie 2). While testing under anaesthesia did not enable tracking over time of stimulation efficacy under chronic implantation scenarios, the stability of impedance and recording performance (Fig. 3) supports the application of stimulation for similarly long timepoints. Our results supported that implants like the ones developed could be used for the restoration of hand movement in conditions such as spinal cord injury and stroke.

### Ultraconformable nerve cuffs identify and track changes in C fibre activity in animal models of neuropathic pain

The ability to record nerve activity at high resolutions from awake animals offers unique opportunities for both therapy and research. Having established the capabilities of the nerve cuffs, we sought to employ them to provide novel biological insight into nerve activity changes occurring during the onset of neuropathic pain. Injury to peripheral nerves can result in enhanced nociceptive C fibre activity, leading to pain^42^. While animal models of nerve injury and neuropathic pain exist, there is a lack of characterisation of changes in nerve activity in awake conditions, and insight into physiological changes that can be tracked longitudinally beyond behavioural tasks is limited. These model and tool shortcomings can lead to difficulties in research on the condition and the development of treatments^43^. The ability of the cuffs developed here to record nerve activity in awake animals, and in particular to classify signal velocity and conduction orientation (Fig. 4), suggested they may be a powerful tool to identify and track over time changes in nociceptive C fibre activity following nerve injury.

**Fig. 6:**
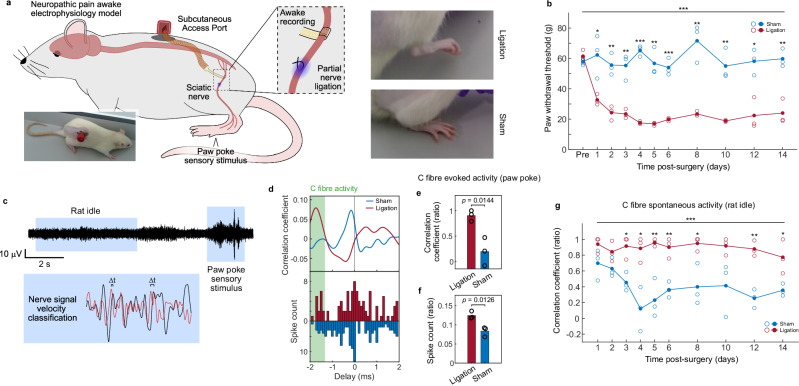
C fibre activity tracking in a partial nerve ligation model of neuropathic pain. **a** Diagram and pictures of the experimental setup. Rats are implanted with a cuff on their sciatic nerve. Animals additionally implanted with a suture to partially ligate the nerve (Ligation) develop hind paw pain as evidenced by their retracted paw posture. Animals with nerve cuffs but without ligation (Sham) do not show this retracted paw posture. Nerve recordings are performed through the implanted cuffs in awake, freely-moving animals. **b** Plot of force applied before rats withdraw their operated hindlimb at various times post-implantation of cuff and ligation. Individual values are shown as open circles; group means are shown as solid circles (*n* = 3 animals). The statistically significant decrease in force threshold corresponds to development of pain. Statistical comparisons by two-way ANOVA (all timepoints) and two-tailed Student’s t-test (individual timepoints). *p < 0.05, **p < 0.01, ***p < 0.001. **c** Depiction of analysis strategy to quantify C fibre activity in awake animals. Zoomed in trace represents recordings from two microelectrodes, one on the proximal and one distal end of the implanted cuff. **d** Representative quantifications of nerve signal distribution based on delay/velocity in an animal with neuropathic pain (blue) and without neuropathic pain (red). Top: cross-correlation value, and bottom: inter-spike interval between microelectrodes at proximal and distal ends of the cuff. Neural signals at velocities identified as corresponding to C fibres (1.0 – 1.5 m/s afferent) are highlighted in green, observed as being higher in neuropathic pain animals. **e, f** Quantification of C fibre activity correlation coefficient and inter-spike interval in neuropathic pain compared to sham control in response to a paw poke of sufficient force to induce paw withdrawal, as ratios to the highest sensory correlation coefficient peak (**e**) or total spike number (**f**). *N* = 3 animals, each corresponding to the average of three Von Frey filament paw probe events. Statistical comparison carried out via two-tailed Student’s t-test. **g** Plot of C fibre activity (correlation coefficient as ratio, similar to (**e**)) in idle animals over different intervals post-implantation. Animals with nerve ligation develop an increase in spontaneous C fibre activity in absence of external stimuli which is preserved throughout the tested period following nerve ligation. Statistical comparisons as in (**b**). Source data for (**b**) and (**e**–**g**) are provided as a Source Data file.

To induce neuropathic pain in rats we made use of the partial sciatic nerve ligation model^44^, which we applied in combination with a single nerve cuff implanted into the same nerve (Fig. 6a). Ligating the sciatic nerve resulted in the robust appearance of neuropathic pain, as characterised by a decrease in the force threshold to induce hind paw withdrawal in the Von Frey test (Fig. 6b, pre-surgery: 59.7 ± 3.6 g, ligation: 23.4 ± 1.7 g, sham: 71.65 ± 12.6 g, mean ± standard deviation at day 8 post-surgery). The implantation of the cuff itself in absence of nerve ligation did not have a significant effect on evoked pain (*p* = 0.4067, one-way ANOVA across all time points and pre-implantation for sham group), indicating that chronic implantation of the cuffs did not impact nerve health.

We paired the neuropathic pain awake behavioural examination with nerve recordings of the affected nerve using the implanted cuff, specifically by employing its unique capability to sort recorded activity by velocity and orientation to selectively analyse slow C fibre activity (Fig. 6c). In response to the application of a probe to the plantar region of the paw (Von Frey testing), we observed a marked increase in C fibre activity (which we defined as 1.0–1.5 m/s afferent signals) in animals that had undergone nerve ligation compared to sham controls (Fig. 6d). This increase was statistically significant both when examining changes in the correlation coefficient (Fig. 6e, ligation: 0.91 ± 0.11, sham: 0.20 ± 0.28, ratio to highest sensory correlation peak) and in spike intervals (Fig. 6f, ligation: 0.13 ± 0.01, sham: 0.08 ± 0.01, ratio to all spikes) at this velocity range.

Having characterised increases in nociceptive C fibre activity during paw retraction in response to Von Frey testing, our study then aimed to investigate whether variations in C fibre activity are also present during intervals not evaluated by existing behavioural neuropathic pain models. Specifically, we sought to characterise changes during periods of idle behaviour when animals are inactive, in absence of any external sensory stimulus (i.e. spontaneous pain). Awake nerve recordings identified an overall increase in C fibre activity when animals were idle which was present throughout the 14 days tested following ligation (Fig. 6g, ligation: 0.95 ± 0.09, sham: 0.40 ± 0.26, ratio to highest sensory correlation peak at day 8 post-surgery). No difference between ligation and sham groups were detected when spike interval, rather than correlation coefficient, was used to characterise C fibre activity (Supplementary Fig. 9, ligation: 0.10 ± 0.02, sham: 0.12 ± 0.02, ratio to all spikes at day 8 post-surgery). Notably, animals in the sham group also exhibited a higher degree of spontaneous C fibre activation during the first two days post-surgery, before activity decreased to a lower baseline (Fig. 6g). We associate this increased C fibre activation with nociceptive post-operative pain, rather than neuropathic pain. Nociceptive pain accompanying the surgical procedure itself, while not leading to decreases in withdrawal threshold (Fig. 6b), is commonly observed in animal surgical procedures and a point of animal experimentation refinement.

## Discussion

Existing nerve interfaces either use a high-invasive penetrating design to connect multi-electrode arrays with multiple portions of the nerve or use less invasive designs cuffing around whole nerves. Despite the long-term advantages of low-invasive designs, cuffs have traditionally been produced using rigid materials and bulky constructions^1,2,8^, resulting in generally low interface qualities and recording SNR. Stimulation with cuffs has been comparatively more successful, seeing use in human patients in the form of therapies such as vagus nerve stimulation^6,7^. However, clinical-use cuffs typically stimulate the entire nerve^7^, therefore limiting the scope for new applications. Nerves contain multiple fascicles innervating different targets, and within these different fibre types, all representing distinct physiological roles. While some studies have developed cuffs with electrode arrays designed to stimulate portions of the nerve, these have relied on the use of larger nerves with more limited therapeutic relevance where some degree of control is easier to achieve, such as the sciatic nerve to control leg muscles^45,46^ or slow deformation of larger nerves to spread out fascicles inside non-circular cuffs^47^.

The nerve cuffs developed in this work combined low-impedance microelectrode arrays with a low profile and conformable design to achieve recording and stimulation at sub-nerve resolutions previously unachieved by nerve cuff implants. From a recording perspective, the cuffs were able to record neural activity unique to certain points on the circumference of the nerve. The nerves of the brachial plexus to which the cuffs interfaced innervated synergist muscles and adjacent skin dermatomes, making interpretation of recorded sub-nerve neural activity difficult. However, we observed that most neural spikes were recorded by either one or two of the three or four microelectrodes surrounding each nerve. As the median, ulnar and radial nerves in rats typically have two to four fascicles each^35^, it seems likely that the sub-nerve resolution recordings correspond to fascicle-specific activity. From a stimulation perspective, the cuffs could stimulate nerves of the forearm with sufficient resolution to not only generate different movements based on the cuff location of the microelectrode (indicative of sub-nerve stimulation resolution) but also produce physiologically meaningful movements such as wrist and finger extension (object release) and flexion (object grasp). Hand movement controlled via nerve stimulation is an active topic of research with applications in central nervous system injury conditions such as spinal cord injury and stroke. Recent advances using a penetrating neuromodulating interface (Transverse Intrafascicular Multichannel Electrode - TIME) have reproduced hand movements such as wrist and finger flexion and extension in non-human primates^15^. Albeit in rodents, the cuffs developed in this work were also capable of producing a comparable range of hand movements despite their less invasive design.

The nerve cuffs were also able to extract conduction velocity and direction from nerve recordings from awake freely-moving animals, a feature which to our knowledge has not been previously reported. Action potential conduction through axons occurs at velocities ranging from approximately 0.4 to 120 m/s, with different types of axon fibres having different conduction velocities. As different nerve fibre populations are associated with different physiological and pathological roles^48^, a method to selectively identify the activity of specific velocities is a highly valuable research and clinical tool. Methods to sort recorded nerve activity based on conduction velocity and direction have long existed^36^ and have mostly been used and validated in animals under anaesthesia to analyse the velocity of electrically-evoked compound action potentials^38,39^. However, the lack of implantable devices with high quality recordings and precise electrode positioning has prevented their translation to more physiologically-relevant awake models. The high interface quality and compact design of the cuffs developed in this work allowed us to implement this feature. Our devices recorded action potential conduction velocities in the expected physiological range. Earlier velocity analysis methods often relied on the addition of individual action potentials with varying amounts of delay, producing as a readout the maximum amplitude of the *delay-and-add* trace^36,37,39^. This method was not well suited for velocity sorting of large volumes of bidirectionally transmitted spikes from awake animals, leading us to develop two new analysis methods. Although the experimental design and behavioural setup of our study precluded the selective manipulation of specific nerve fibre populations, the consistency between the outcomes of both analysis methods and their alignment with established velocities of known fibre populations^41^ support the efficacy of the velocity sorting capabilities of our cuffs.

We employed the developed nerve cuffs, and in particular their ability to characterise nerve signal conduction velocity and direction, to explore changes in C fibre activation in neuropathic pain. Partial nerve ligation is a commonly employed model of neuropathic pain, resulting in decreases in paw withdrawal threshold similar to that we observed^49^. Despite the high burden of neuropathic pain on society^42^, limitations of animal models remain a barrier to the development of therapies^43^. We demonstrated the potential of the developed cuffs as research tools by providing the first characterisation of changes in C fibre activation in awake animals – a key pathophysiological event driving the appearance of pain in humans. Furthermore, our results demonstrate that this model of pain exhibits suitable characteristics beyond those previously described. While allodynia and evoked pain are well characterised and easy to test models of neuropathic pain, spontaneous pain is more difficult to quantify despite being a core component of neuropathic pain in humans^43,50,51^. The developed nerve cuffs provide such a quantifiable characterisation of spontaneous C fibre activation in awake animals, showing that pain signalling is present without a sensory stimulus and extending the validity of this neuropathic pain research model.

One of the main challenges of peripheral nerve interfaces is chronic stability. Unlike the CNS, nerves are located in highly dynamic environments characterised by large movements and forces and are not encased in protective bone structures. These properties can make breakages in implants a common occurrence and exacerbate the tissue damage and FBR generated due to tissue-implant mechanical mismatch^8^. The use of robust thick-film fabricated designs has enabled whole-nerve stimulation in cuffs lasting years^52^ and the more fragile, microfabricated penetrating nerve interfaces have also been used for stimulation in humans for periods of months^53^. Long-term recordings are much more difficult to achieve as they heavily rely on the presence of a high-quality tissue-electrode interface, which can quickly degrade due to chronic tissue inflammation and FBR. Traditional cuff electrodes generally perform poorly in nerve recordings due to their overall worse nerve-electrode interface, with few applications making use of them outside of animal models under acute anaesthesia^2,8,39,40,54^. Penetrating devices perform better. Slanted Utah electrode arrays have been used to record nerve activity in humans for up to 27 days, albeit with some progressive decrease in the number of action potential-recording electrodes^55^ and with the development of chronic inflammation, which could pose a challenge to long term use^56^. Our conformable cuffs achieved stable recordings in awake animals for the 21 days over which we performed recordings, with minimal loss of electrodes, negligible change in impedance after implantation and almost no formation of fibrotic scar tissue. Moreover, the cuff design provides a number of advantages compared to penetrating devices with regard to maintaining a stable recording interface. The proximity of penetrating devices to axons makes them more sensitive to the build-up of fibrotic tissue at the interface, in contrast to cuffs designed to record through a ~ 50 μm fibrous epineurium. Cuffs also transfer forces to the nerve through the epineurium – a collagen-rich tissue responsible for protecting the nerve – whereas penetrating devices interact with and cause chronic inflammation in the much more fragile endoneurium where axons are located.

Overall, the developed nerve cuffs serve as a tool for a wide range of applications. The ability to record chronically while identifying active nerve fibre populations in awake animals provides a new avenue to study the physiology of nerves and innervated structures, particularly when combined with fascicle-specific recording and neuromodulation enabled by this technology. Clinically, the developed technology may not only see use in the control of hand movement but may also serve as a less invasive and more chronically-stable neuromodulation tool than penetrating devices, potentially also enabling the clinical use of nerve recordings for diagnostic or closed-loop applications.

## Methods

### Device fabrication

Implants were fabricated using photolithographic techniques for flexible electronic devices^12^. PaC, from dichloro-p-cyclophane (Specialty Coating Systems, Indianapolis, IN, USA) was deposited (Specialty Coating Systems Labcoater – Specialty Coating Systems, Indianapolis, IN, USA) at a 2 μm thickness onto a Si wafer. Gold tracks were patterned (MA/BA6 or MJB4 mask aligner – Süss Microtec, Garching, Germany) using AZ 5214E photoresist and AZ 400 K developer or using AZ nLOF 2035 photoresist with AZ 726 MIF developer (Microchemicals GmbH, Ulm, Germany). A 10 nm Ti adhesion layer followed by a 100 nm Au layer was deposited (Lesker e-beam Evaporator – Kurt J. Lesker Company, Jefferson Hills, PA, USA) onto the PaC layer, and a gold lift-off was performed using acetone. A second layer of PaC was deposited, insulating the gold tracks. The outline of the device was patterned using AZ 10XT 520 cP (Microchemicals GmbH, Ulm, Germany) photoresist with AZ 726 MIF developer to create an etch mask. The outline was etched using reactive ion etching (PlasmaPro 80 Reactive Ion Etcher (RIE) – Oxford Instrument, Abingdon, UK), using 8 sccm CF_4_, 2 sccm SF_6,_ and 50 sccm O_2_ at 60 mTorr. Miro-90 Concentrated Cleaning Solution (Cole-Parmer, Vernon Hills, IL, USA) was spin-coated onto the devices as an anti-adhesive layer before a third layer of 2 μm of PaC was deposited onto the wafer. The electrodes and connector pads were patterned and etched in the same manner as the device outline. Electrodes were then spin-coated with a PEDOT:PSS coating, consisting of 5 v/v% ethylene glycol, ~30 µL of DBSA, 1 v/v% GOPS (Sigma Aldrich, St. Louis, MI, USA), remainder PEDOT:PSS (Clevios PH 1000 – Heraeus, Hanau, Germany). After coating, the anti-adhesive PaC layer was peeled off, leaving discretely-coated electrodes. Finally, implants were removed from the Si wafer, and an FFC (Mouser Electronics, Mansfield, TX, USA) was bonded (Finetech Bonder Fineplacer® System – Finetech GmbH, Berlin, Germany) onto the connector using anisotropically conductive film (5 μm particulate) (3TFrontiers, Singapore) to create the implant.

### Surgical implantation

All animal procedures were carried out in accordance with the UK Animals (Scientific Procedures) Act, 1986. Work was approved by the Animal Welfare and Ethical Review Body of the University of Cambridge, and was approved by the UK Home Office (project licence numbers PFF2068BC and PP5478947). Experiments were conducted on Sprague Dawley (nerve stimulation, ~200 g, ~9 weeks of age) or Lewis (chronic nerve electrophysiology recordings, ~150 g, ~9 weeks of age) rats (Charles River, UK). Rats were group-housed in individually ventilated cages with ad libitum access to food and water for the duration of the study. Animal sex was not considered in the design of this study given that device performance was the main purpose of the study.

Surgical implantation of devices was carried out under isoflurane anaesthesia (2.25% v/v in medical oxygen). Body temperature was monitored and maintained using a thermal blanket. For non-recovery (nerve stimulation) experiments, an incision was made in the ventral portion of the arm. The bundle of radial, ulnar and median nerves was accessed between the humerus and the triceps muscle at the upper-arm level. The three cuffs of the device were implanted by wrapping around each of the three nerves, and sealed shut using a drop of Kwik-Cast silicone (World Precision Instruments) applied between the locking tab and the PaC connector. Care was taken not to allow silicone to flow into or around the cuff portion of the implant.

For chronic electrophysiology recording experiments, the nerves were accessed at a similar height dorsally, and cuffs were similarly implanted. The PaC and FFC connectors were routed subcutaneously to the head of the animal, and held in place using a 6-0 suture (Ethilon Nylon, Ethicon). The PaC portion of the connector was reinforced with a thin layer of Kwik-Cast, applied after cuff implantation. Additional length of PaC connector was folded over the area of implantation to allow for stretching of the implant during movement. The connector was then routed to a custom 3D-printed headcap, which was secured to the head of the animal using surgical cement (Unifast Trad, Billericay Dental Supply Co Ltd; Super Bond C&B, Prestige Dental Products Ltd) and drilled steel screws (M0.8, US Micro Screw). An additional screw was drilled above the cerebellum and connected to using a steel wire to use as an electrophysiology ground. This provided a clean ground connection without cardiac signal contamination and risk of movement artefacts that was well suited to the shoulder implantation location employed in forearm nerve implants. The FFC and ground wire were connected to a custom PCB integrated into the headcap, which provided the connections to perform electrophysiology in awake animals. During implantation, additional length of FFC cable was left within the headcap to allow the cable to stretch with the body during movement without placing tension on the implant or tissue.

While the FFC is not stretchable, we addressed the need for extension of the cable during animal movement by leaving an additional “slack” length of cable within the headcap. This allowed the FFC to effectively stretch with the body if necessary, without putting tension on any end of the implant (nerve or connector). We did not experience major damage to the subcutaneous space in which the FFC was placed, likely due to this body cavity being relatively open and allowing the implant to move without pressing on tissues. The only exception to this was a single animal, in which a sharp corner of the FFC began damaging the skin within the subcutaneous pocket, leading us to cull the animal at day 14 post-implantation. The only part of the implant which was routed around and through muscle was the parylene portion. While this material is not stretchable, we left abundant slack length of connector to allow the device to effectively stretch during movement.

For the chronic FBR immunohistochemistry study, the radial nerve was accessed ventrally at the same height, and a cuff consisting of either a medical-grade PDMS tube (Syndev, 0.63 mm inner diameter, 1.19 mm outer diameter, 228-0254 VWR), implantable cannula PE tube (0.6 mm inner diameter, 1.1 mm outer diameter, 504280, World Precision Instruments), or a PaC cuff was implanted. The tube implants were produced by slicing open a 2 mm-long portion of tube and allowing it to wrap around the nerve. The PaC cuffs consisted of median or radial cuffs from nerve cuff devices, cut at the PaC connector portion of the device. These were implanted by wrapping around the nerve and sealed with a drop of Kwik-Cast silicone between locking tab and PaC connector portion of the implant. Care was taken to not allow silicone to flow into or around the cuff portion of the implant.

For neuropathic pain awake recording experiments, a modified implant containing a single cuff with identical electrode dimensions was implanted into the right sciatic nerve of anaesthetised animals, accessed dorsally. The FFC was routed through the back of the animal to a modified vascular access button placed in the mid-back of the animal (VABR1B/22, Instech Laboratories). The steel ground wire was placed underneath the skin of the animal, below this access port. During the implantation of the cuff, a suture (9-0 Ethilon nylon suture, Ethicon) was tied around half of the sciatic nerve to induce pain, similar to described partial nerve ligation protocols^44,49^. Sham animals had a cuff implanted but no ligation was performed.

In chronic implantation, surgeries (FBR and electrophysiology recordings), incisions in the arm were sutured closed (5-0 Monocryl sutures, Ethicon) and animals were recovered from anaesthesia. Analgesia was provided for two days following surgery (Metacam, oral suspension), and were kept under post-operative observation for three days following surgery.

### Immunohistochemistry

Four weeks post-implantation animals were humanely killed by rising CO_2_ concentration. The implanted nerves were accessed and dissected out together with their implant, and fixed in formalin overnight at 4 °C. The cuffs were then carefully removed under a dissection microscope and the nerves were trimmed to keep only the portion which had been surrounded by a cuff. The nerve samples were then transferred into phosphate buffered saline with 30% w/w sucrose for cryoprotection, mounted in optimal cutting temperature (OCT) compound (361603E, VWR), and sectioned in a cryostat (CM1900, Leica) into 10 μm-thick cross-sections of the nerves.

For staining, sections were blocked in 5% donkey serum (D9663, Sigma) in phosphate buffered saline and 0.1% sodium azide for 1 h at room temperature. Anti-αSMA (1/100 dilution, ab7817, Abcam) and Anti-β3 tubulin (1/1000 dilution, ab18207, Abcam) antibodies in a blocking medium were added for 3 h at room temperature in the dark. This was followed by incubation in secondary antibodies (Donkey anti-Rabbit IgG (H + L) Highly Cross-Adsorbed Secondary Antibody, Alexa Fluor 555; and anti-Mouse, Alexa Fluor 488; Invitrogen) for 3 h at room temperature in the dark. Finally, sections were incubated in Vector TrueVIEW Autofluorescence Quenching Kit (Vector Laboratories) for 3 min at room temperature, mounted, and imaged in a Axioscan Slide Scanner (Zeiss). Between incubation steps, sections were washed three times, 10 min per wash, with a blocking medium.

Analysis was carried out using custom scripts in MATLAB (Mathworks, 2016b) and Fiji (v1.48, National Institutes of Health, USA) in 20X magnification images. These produced a stain intensity profile of αSMA as a function of depth into the nerve, starting from the outermost portion of the circumference where the implant was located as defined by the user. Intensity was normalised into a ratio relative to αSMA intensity in the middle of the nerve cross-section of each nerve. Statistical analysis and data plotting was carried out in MATLAB (Mathworks, R2016b).

### Tensile tests

For mechanical testing of PaC, a PaC film was deposited onto a Si wafer as above at 6 μm thickness. PaC films were cut (Silhouette Portrait – Silhouette America, Inc., Lindon, UT, USA) into dog-bone mechanical testing samples. Samples were tested to failure (Tinius Olsen 1 ST – Tinius Olsen, Horsham, PA, USA) at a rate of 10 mm/min. Stress and strain were tabulated and used to calculate the elastic modulus. For mechanical testing of PE and PDMS, tubes of each material were tested as is within the elastic regime at a rate of 0.8 mm/min. Stress and strain were tabulated and used to calculate the elastic modulus.

### Nerve awake electrophysiology recordings

Electrophysiology recordings were carried out from rats at various timepoints post-implantation. Recordings were performed through an Intan RHS stim/recording system (Intan Technologies) connected to the PCB on the rat headcaps, amplified (x 192), bandpass filtered between 1 Hz and 7.5 kHz and sampled at a rate of 30 kHz. The cerebellar screw connected to this PCB was used as a ground for these recordings. Impedance measurements were performed using this same system and setup. Recordings were performed while rats walked through a transparent acrylic tunnel with an open top (500 mm long by 80 mm wide). Animals were video recorded during these tasks using a high frame-rate camera (GoPro Hero 6 Black, 120 fps). The walking task was repeated five times per rat per timepoint. The electrophysiology and video recordings were synchronised using an LED driven by the electrophysiology system. Swing and stance portions of the rat movement were identified manually from the recorded videos. Recording experiments were performed over 21 days post-implantation. One rat had to be culled 14 days post-implantation due to skin damage caused by the device FFC connector. Paw dexterity was also evaluated in animals by approaching them to a narrow bar. Animals naturally reached out to this bar and attempted to grip it with their implanted limb. Performance was recorded and scored as Miss (animals missed the bar or failed to reach out), Lean (animals reached, touched, and held their paw on the bar but failed to grip it), or Grip (animals reached out, touched, and gripped the bar). During the task the non-implanted forelimb was held to restrict its movement and ensure animals used their implanted limb exclusively.

For neuropathic pain recordings, animals were placed in a transparent acrylic box approximately 200 by 120 mm in size with an open top. Their implant FFC and ground wire were accessed by removing the cap from the subcutaneous access port, and connected directly to the Intan RHS system through a custom PCB. Electrophysiology recordings were performed over approximately two minutes while the animals remained stationary. Following this, the animals were placed within these same boxes over a suspended grid and the force withdrawal threshold for their right hind paw was obtained using an electronic Von Frey system (EVF4S, Panlab). The system calculated the maximum force at which animals withdrew their paw when a plastic tip probe was used to poke the planar surface of the paw from underneath. For each animal and timepoint, four force withdrawal threshold recordings were performed and averaged. Idle electrophysiology and force withdrawal threshold recordings were performed over two weeks after implantation. Force withdrawal thresholds were also obtained for animals the day prior to implantation. In addition to these recordings, an electrophysiology recording session was performed at day 8 post-implantation while animals were being tested for paw withdrawal, producing the paw poke sensory stimulus recording dataset.

### Electrophysiology recording analysis

Electrophysiology analysis and data plotting was performed in MATLAB (Mathworks, R2016b) and Python. Recorded traces were imported and bandpass filtered using a 4th order Butterworth filter with cutoff frequencies 300 and 2000 Hz. For each microelectrode, recordings were converted into a bipolar configuration through signal subtraction where the electrode used as a reference depended on the analysis being carried out. For whole nerve analysis (Fig. 3), the whole nerve electrode from the corresponding cuff of each microelectrode was used. For waveform sorting, neural events (‘spikes’) used as input to the spike sorting algorithms were identified using an adaptive threshold based on the smallest of constant false-alarm rate (SO CFAR) filter^57,58^, with the parameters (window duration of 150 ms on each side, guard cell duration of 10 ms on each side, and threshold level of 3 standard deviations from the mean) being heuristically chosen. The extracted waveforms were initially transformed to reduce dimensionality using the uniform manifold approximation and projection (UMAP) method. The new 3D space corresponding to the waveforms was used as input to an unsupervised clustering algorithm. The K-Means algorithm was used to keep consistency among trials on the number of identified clusters (heuristically set to 5 after preliminary analysis). Finally, a sliding window of 1 s was used to compute the spike rate of the clusters within each window, and the evolution of the metric over time was plotted (see Supplementary Fig. 2). SNR from recordings was calculated as the ratio between the variances of a randomly chosen burst of activity and of a period of no activity (noise), for each of the three cuffs in each implant at every timepoint. Using these same activity and baseline periods, root mean squared values for baseline and mean spike amplitude for activity were calculated (spikes detected using a threshold of 2.5 times the noise root mean squared).

In sub-nerve resolution (Fig. 4) nerve recording selectivity analysis, bipolar referencing was carried out between each microelectrode and the average of the other microelectrodes within that same ring. Microelectrodes within the same column in the array were deployed as a ring around the cuffed nerve when implanted. Co-detection analysis was carried out by first performing spike detection over all the referenced recordings from microelectrodes within the same ring (detection of peaks above 6.5 μV and with a minimum distance of 0.5 ms), and second by analysing whether each identified spike had an equivalent spike in any other channel within that same ring. A spike was considered to be co-detected when the peak of the spike is within 0.5 ms across two or more channels. Depending on whether the spike was co-detected across four (in case of the radial nerve cuff), three, two microelectrodes, or uniquely recorded in one microelectrode, it was categorised as “four channels”, “three channels”, “two channels”, or “single channel”. This analysis was performed for every spike in each microelectrode recording, meaning that for example a spike co-detected across three channels was counted three times. Single channel-recorded spikes were also categorised by the microelectrode within the ring from which they were recorded.

Spike delay analysis was carried out by comparing recorded signals from two bipolar-referenced microelectrodes from rings 2 mm apart within the array. Analysis was carried out in two ways, both intended for fast processing of large volumes of recorded action potential data. The first implementing a thresholding strategy and the second utilising the entire recorded trace. The first approach implemented a thresholding strategy. Spike detection was carried out over the traces of the two microelectrodes (detection of peaks above 6.5 μV and with a minimum distance of 0.5 ms). For each identified spike in a microelectrode, the spike closest in time in the other microelectrode recording was found and the delay between the two was calculated. The analysis was performed for spikes over both microelectrodes. Spike delays between −2 and +2 ms were grouped into a histogram with bin size 0.1 ms. Spikes outside of this range were discarded. This method made it possible for spikes corresponding to different axons to be erroneously matched and lead to some degree of erroneous co-detection. However, as average spike rates in microelectrodes were of 54.76 ± 9.61 spikes per second, with a spike typically lasting 1 ms, these erroneous co-detections are expected to make up only a small fraction of total analysed spikes. The second approach made use of the entire recorded trace to avoid any velocity data distortion introduced by thresholding. The correlation coefficient was calculated between the two microelectrode traces when delays ranging from −2 to +2 ms were introduced. Results from the two analyses were plotted, and delays in spike/correlation were converted to conduction velocity and direction of neural activity using the distance and position on the nerve of the two microelectrodes.

The neuropathic pain electrophysiology recording dataset was analysed using the same spike delay analysis specified above over three paw poke events with a Von Frey probe (paw poke sensory stimulus) or a 5 to 10 s period (rat idle). C fibre quantification was then performed by selecting correlation coefficient or spike counts in the range of −2 to −1.33 ms (corresponding to 1.5 to 1.0 m/s sensory signals in a 2 mm microelectrode spacing cuff). Correlation coefficient quantification was performed by calculating the ratio between the highest correlation coefficient value in this C fibre range to the highest correlation coefficient among the sensory signals (between −2 and 0 ms delays). Spike count quantification was performed by calculating the ratio between all spikes in this C fibre range to all spikes analysed (between −2 and +2 ms delays).

### Nerve stimulation

Nerve stimulation was carried out on animals under isoflurane anaesthesia (1.75% v/v in medical oxygen) using an RHS stim/recording system (Intan Technologies). The system was connected to the implanted nerve cuffs with 32 microelectrodes, and used a larger electrode present in all three cuffs as a ground. Nerve stimulation was carried out through individual microelectrodes via trains of 100 biphasic pulses (100 μs and 100 μA per phase, 10 ms period). The resulting muscle contractions and movements of the implanted paw were recorded using a high frame rate camera (GoPro Hero 6 Black, 120 fps) from two angles (top and side of paw).

Analysis of movements and movement kinematics as well as data plotting was carried out in MATLAB (Mathworks, R2016b). Movements were classified by hand blindly (experimenter blinded to what movement corresponds stimulation through which microelectrode) based on the closest match between a series of predefined movements. These predefined movements were finger extension or flexion, wrist extension or flexion, radial or ulnar deviation, and paw pronation or supination. Recorded movements were identified as producing either a single of these predefined movements, a combination of two of these movements, or no movement at all. For kinematic analysis, video recordings were imported and position markers were placed over the paw for the movement produced by each microelectrode stimulation, as well as the paw resting position. Markers were placed on the tip of the middle digit (D3) and middle knuckle (top view video) and tip of thumb digit (D1), middle digit (D3) and little digit (D5) (side view video). X and Y positions of the markers were normalised to additional markers used as position origin on the base of the wrist (both top and side view). The position kinematics were processed by principal component analysis, and plots of the first and second, as well as first and third principal components, were produced.

### Reporting summary

Further information on research design is available in the Nature Portfolio Reporting Summary linked to this article.

## Supplementary information

Supplementary Information

Peer Review File

Description of Additional Supplementary Files

Supplementary Movie 1

Supplementary Movie 2

Reporting Summary

## Source data

Source Data

## Data Availability

All data supporting the findings of this study are available within the article and its supplementary files. Any additional requests for information can be directed to, and will be fulfilled by, the corresponding authors. Source data are provided with this paper. The main data generated in this study and supporting its results have also been deposited in the Figshare database under accession code 10.6084/m9.figshare.21892986 (ref. ^59^). Source data are provided with this paper.

## Code availability

Custom scripts used to carry out stain, electrophysiology and kinematic analysis are available in the following repository: 10.5281/zenodo.7544535.
