# Supplementary material for: Ultraconformable cuff implants for long-term bidirectional interfacing of peripheral nerves at sub-nerve resolutions
Source: Nat Commun. 2024 Aug 30;15:7523. doi: 10.1038/s41467-024-51988-1 (PMC11364531; doi:10.1038/s41467-024-51988-1)
Supplement: Supplementary file 1 — Supplementary Information [file 41467_2024_51988_MOESM1_ESM.pdf]

Supplementary Information for

Ultraconformable cuff implants for long-term bidirectional interfacing of peripheral nerves at sub-nerve resolutions

Alejandro Carnicer-Lombarte[1], Alexander J. Boys[2], Amparo Güemes[1], Johannes Gurke[1,3], Santiago Velasco-Bosom[1], Sam Hilton[1], Damiano G. Barone[1,4,*], George G. Malliaras[1,*]

[1] University of Cambridge, Electrical Engineering Division, 9 JJ Thomson Ave, Cambridge CB3 0FA, United Kingdom.
[2] University of Cambridge, Department of Chemical Engineering and Biotechnology, Cambridge CB2 0QQ, United Kingdom.
[3] University of Potsdam, Institute of Chemistry, Karl-Liebknecht-Str. 24-25, 14476 Potsdam, Germany.
[4] University of Cambridge, School of Clinical Medicine, Department of Clinical Neurosciences, Cambridge Biomedical Campus, Cambridge CB2 0QQ, United Kingdom.

* Corresponding authors: dgb36@cam.ac.uk, gm603@cam.ac.uk

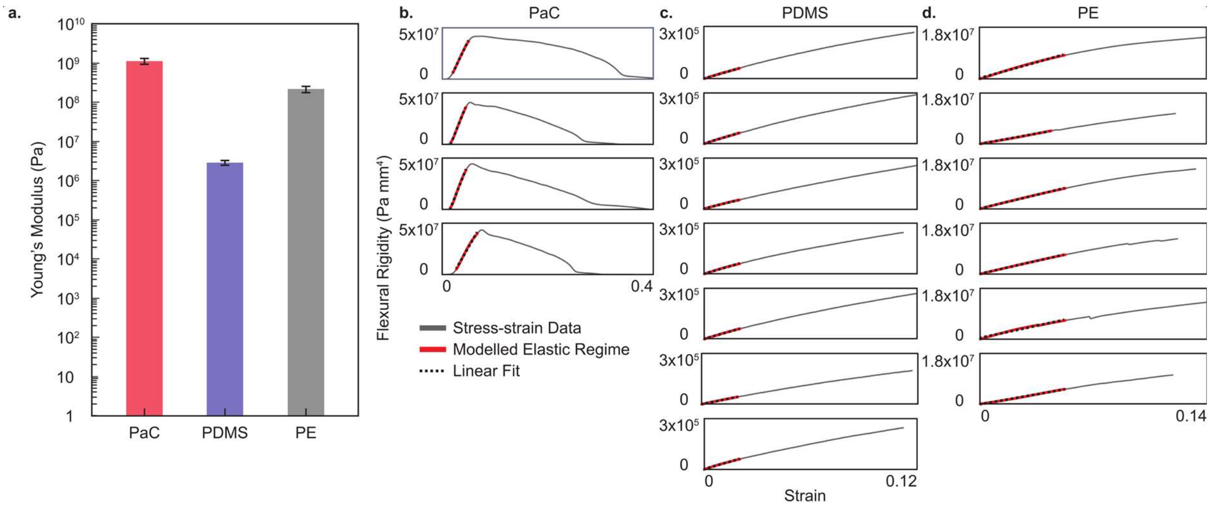

e. Moment of Inertia

Moment of Inertia for a tube:

$$I = \frac{J_0}{2}; I = \text{Moment of Inertia}, J_0 = \text{Polar Moment of Inertia}$$
$$J_0 = \int_A r^2 dA; dA = 2\pi\rho d\rho \text{ (for a circle)}$$
$$J_0 = \int_{r_i}^{r_0} \rho^2 2\pi\rho d\rho$$
$$J_0 = \frac{2\pi\rho^4}{4}\Big|_{\rho=r_i}^{r_0}$$
$$J_0 = \frac{\pi}{2}(r_0^4 - r_i^4)$$
$$I = \frac{\pi}{4}(r_0^4 - r_i^4)$$

Moment of Inertia for a flat plate:

$$I = \int_A y^2 dA; dA = dxdy$$
$$I = \int_0^h \int_0^b y^2 dxdy$$
$$I = \int_0^h y^2 b dy$$
$$I = \frac{by^3}{3}\Big|_{y=0}^{h}$$
$$I = \frac{bh^3}{3}$$

Moment of Inertia for a cylinder:

$$I = \frac{J_0}{2}; I = \text{Moment of Inertia}, J_0 = \text{Polar Moment Inertia}$$
$$J_0 = \int_A r^2 dA; dA = 2\pi\rho d\rho$$
$$J_0 = \int_{r_i}^{r_0} \rho^2 2\pi\rho d\rho$$
$$J_0 = \frac{2\pi\rho^4}{4}\Big|_{\rho=0}^{r}$$
$$J_0 = \frac{\pi}{2} r^4$$
$$I = \frac{\pi}{4} r^4$$

Parameters:

PaC:

$E = 1.13\text{x}10^9$ Pa
$r_0 = 0.3$ mm
$r_i = 0.296$ mm
$h = 0.004$ mm

PDMS:

$E = 2.86\text{x}10^6$ Pa
$r_0 = 0.595$ mm
$r_i = 0.315$ mm
$h = 0.28$ mm

PE:

$E = 2.17\text{x}10^8$ Pa
$r_0 = 0.55$ mm
$r_i = 0.3$ mm
$h = 0.25$ mm

Nerve:

$E \approx 60$ Pa
$r = 0.3$ mm

Flexural Rigidity (EI):

PaC:

$I_{tube} = 3.33\text{x}10^{-4}$ mm^4
$I_{plate} = 2.13\text{x}10^{-9}$ mm^4
$EI_{tube} = 3.76\text{x}10^5$ Pa mm^4
$EI_{plate} = 2.41$ Pa mm^4

PDMS:

$I_{tube} = 9.07\text{x}10^{-2}$ mm^4
$I_{plate} = 7.32\text{x}10^{-4}$ mm^4
$EI_{tube} = 2.59\text{x}10^5$ Pa mm^4
$EI_{plate} = 2.09\text{x}10^3$ Pa mm^4

PE:

$I_{tube} = 6.55\text{x}10^{-2}$ mm^4
$I_{plate} = 5.21\text{x}10^{-4}$ mm^4
$EI_{tube} = 1.42\text{x}10^7$ Pa mm^4
$EI_{plate} = 1.13\text{x}10^5$ Pa mm^4

Nerve:

$I_{cylinder} = 6.36\text{x}10^{-3}$ mm^4
$EI_{cylinder} = 0.38$ Pa mm^4

Supplementary Fig. 1 | Mechanical testing and flexural rigidity calculations different nerve cuff materials and nerve. a, Young's modulus distribution for each material. **b-d,** testing curves for (**b**) PaC, (**c**) PDMS and (**d**) PE. **e,** Flexural rigidity calculations. Source data for (a) are provided as a Source Data file.

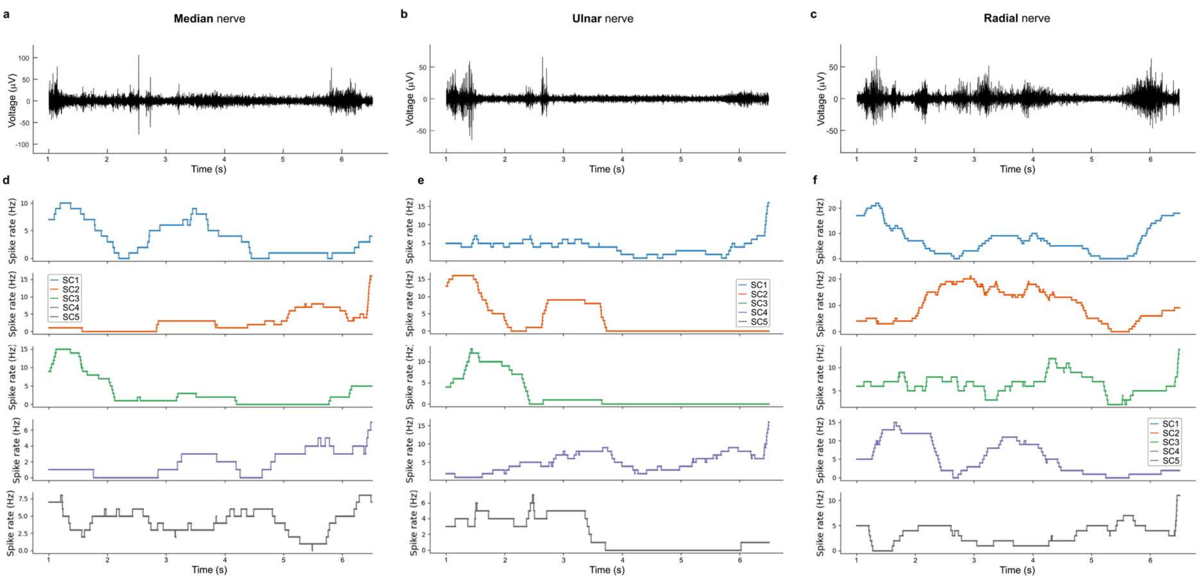

Supplementary Fig. 2 | Temporal evolution of spike clusters among the three nerve cuffs. a-c, Simultaneous neural recordings from median (**a**), ulnar (**b**) and radial nerves. **d-f**, Spike rates for five spike clusters sorted from recordings in (a-c), with a one second averaging window. Different clusters contribute predominantly to certain activity bursts in whole nerve recording traces. Traces and clusters obtained from an awake rat 3 days post-implantation (same as Fig. 3f-g).

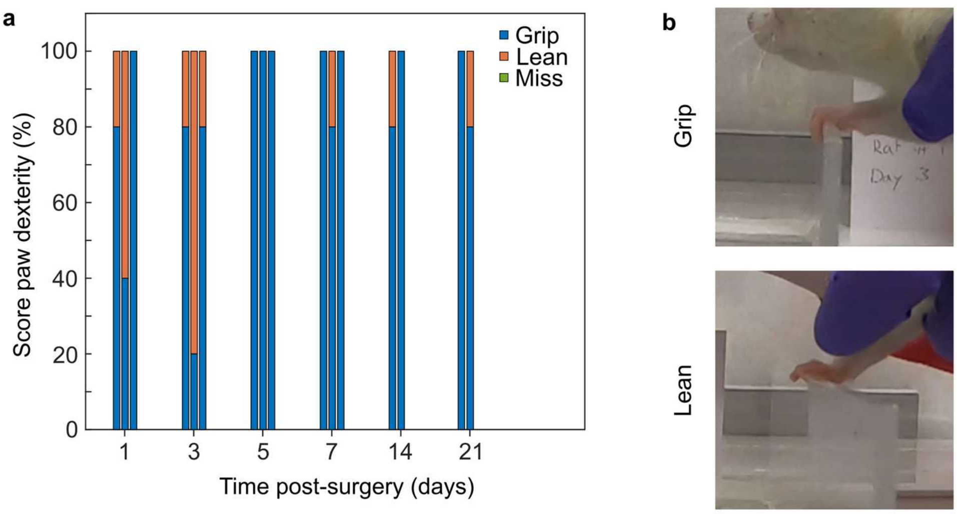

Supplementary Fig. 3 | Movement and dexterity of front paw is not impaired by the presence of the nerve implant. a, Histogram of paw dexterity behaviour scoring for three implanted rats at various timepoints. Task consisting of animals reaching out to a bar was scored according to performance (Miss: animals missed the bar or failed to reach out, Lean: animals reached, touched, and held their paw on the bar but failed to grip it, Grip: animals reached out, touched, and gripped the bar). No misses are observed throughout the implantation period, with only a temporary decrease in gripping observed. **b**, Sample pictures of animals during dexterity testing for Grip and Lean scorings.

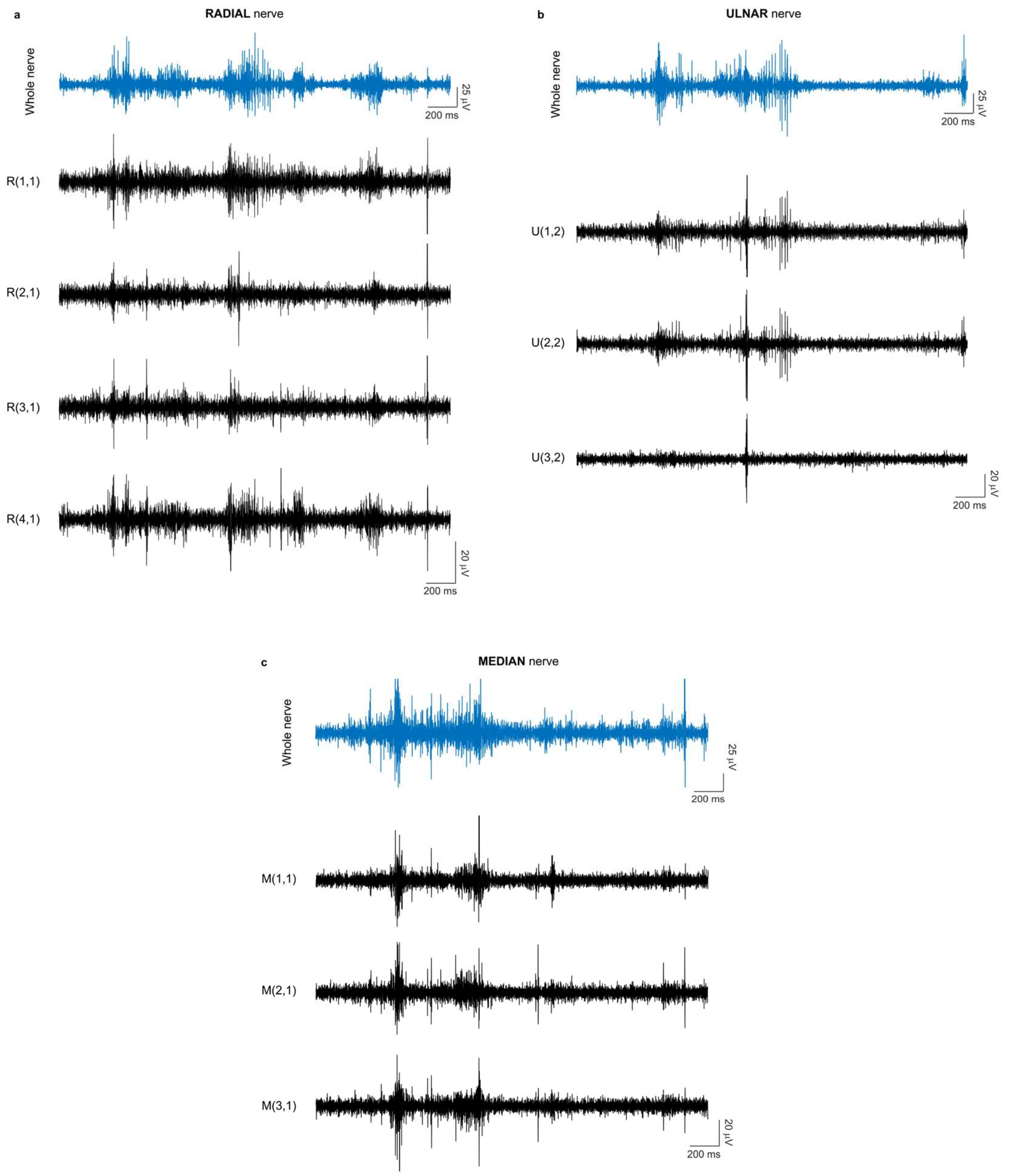

Supplementary Fig. 4 | Cuff recordings along the circumference of radial, ulnar and median nerves. **a**, Recording from whole nerve electrode (top, blue) and from four microelectrodes in a ring along the circumference (bottom, black) of the radial nerve. Microelectrode traces correspond to those on which event thresholding is carried out in Figure 4b. **b-c**, Recordings from whole nerve and ring of microelectrodes from ulnar (**b**) and median (**c**) nerves. All three nerve recordings are obtained simultaneously from the same rat, 3 days post-implantation.

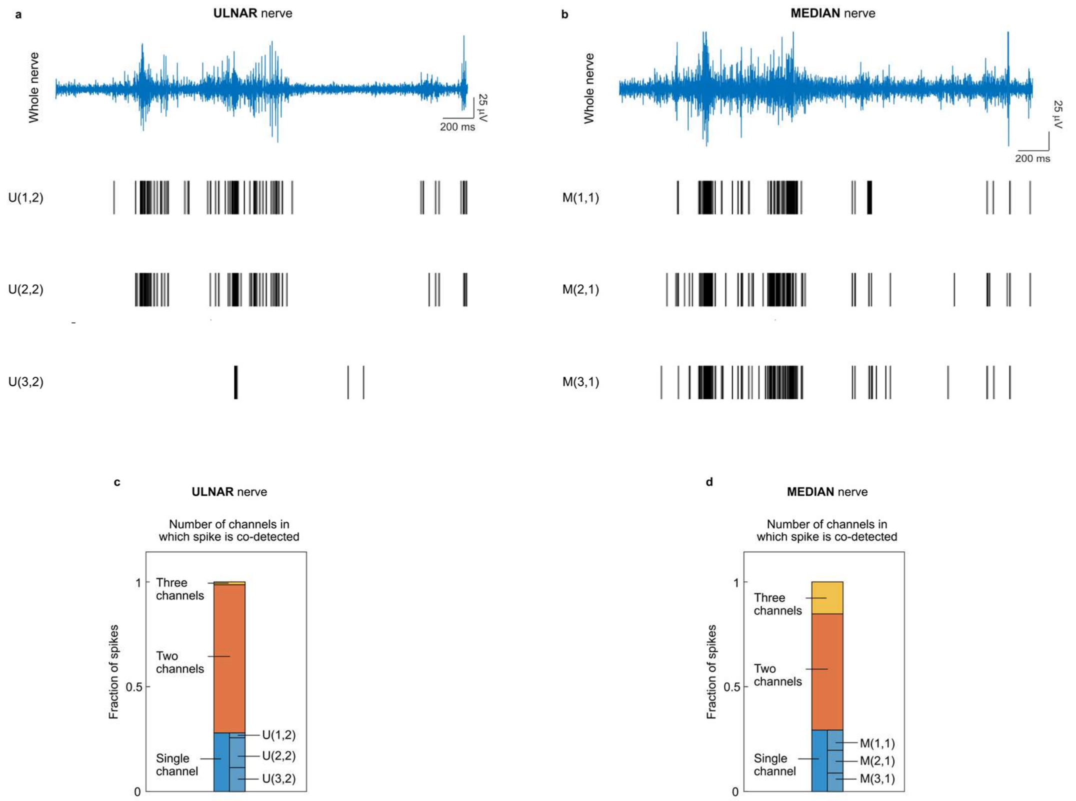

Supplementary Fig. 5 | Circumference sub-nerve recording capabilities of ultraconformable cuff microelectrodes in ulnar and median nerves. a-b, Spike events across three microelectrodes within a ring along the ulnar (**a**) and median (**b**) nerve circumference. The whole nerve recording is provided (blue) for reference. Spike thresholding is carried out over recording traces from Supplementary Figure 2b-c. **c-d**, Quantification of spike coincidence across microelectrodes for activity shown in (a-b). Recordings and coincidence calculation obtained simultaneously from those of Figure 4b-d from an awake rat 3 days post-implantation.

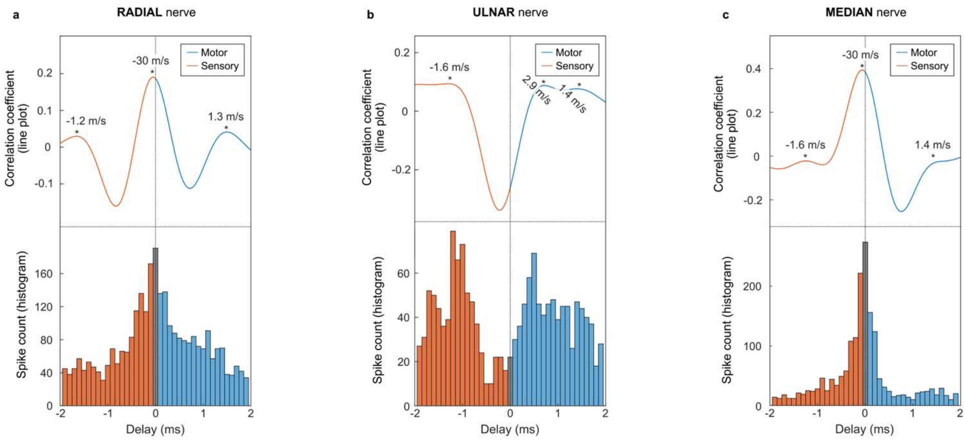

Supplementary Fig. 6 | Nerve activity direction and velocity analysis using longitudinal sub-nerve recording capabilities of ultraconformable cuff microelectrodes. a-c, Quantifications of nerve activity delay between microelectrodes within the most proximal and most distal rings in the array of each cuff, for radial (**a**), ulnar (**b**) and median (**c**) nerves. Top: quantification corresponding to the cross-correlation value between the nerve recordings of the two microelectrodes at varying time delays. Bottom: histogram corresponding to the inter-spike interval between microelectrodes. Analysis identifies different peaks of sensory and motor activity at various values, differing across the three nerves. Analysis performed over a 15-second long recording obtained simultaneously from all three nerves from an awake animal 3 days post-implantation. Recordings used for analysis differ from those used in Figure 4g. Velocities are calculated based on the 2 mm distance between microelectrodes.

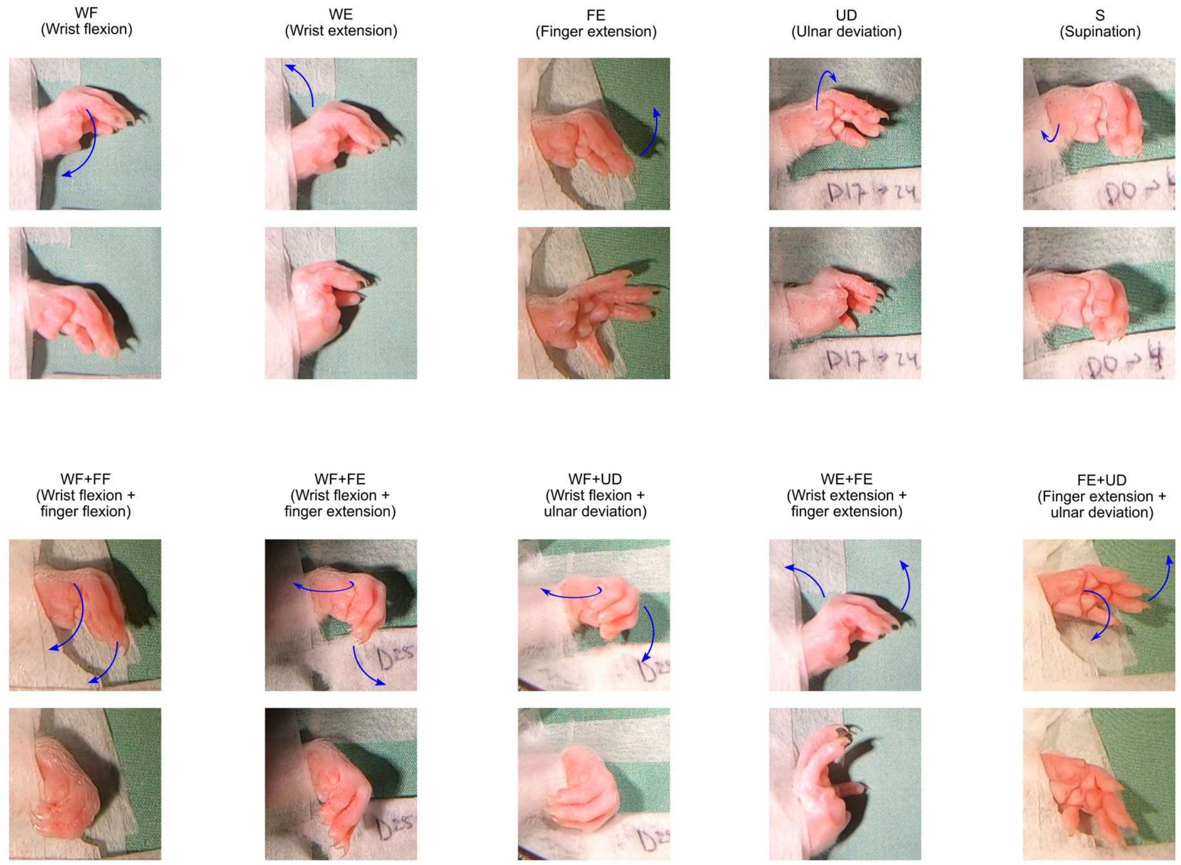

Supplementary Fig. 7 | Picture gallery of paw movements induced by nerve stimulation through ultraconformable cuffs. Pictures before (top) and during (bottom) nerve stimulation. Blue arrow indicates direction of movement. Composite movements (bottom row, indicated by "+" in their name) consist of two simultaneous movements. Movements can occur around both wrist (WF, WE, UD, S) or fingers (FF, FE).

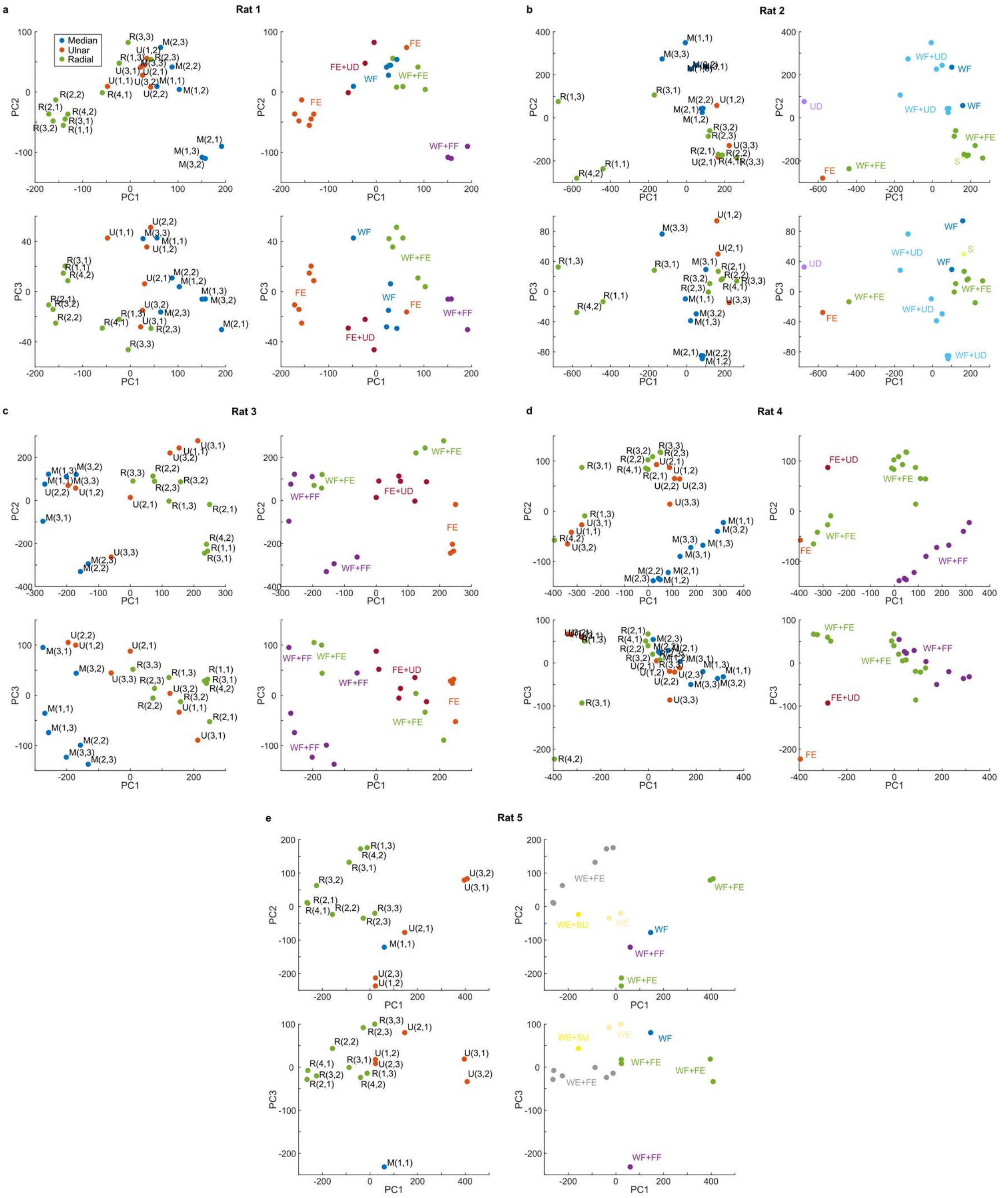

Supplementary Fig. 8 | Analysis of kinematics of movements induced by nerve stimulation through ultraconformable cuffs. a-e, Principal component analysis of kinematics for Rat 1 (**a**), Rat 2 (**b**), Rat 3 (**c**), Rat 4 (**d**) and Rat 5 (**e**). Top panels: plots of principal components 1 against 2. Bottom panels: plots of principal components 1 against 3. Left panels: movements are tagged based on the microelectrode that produced them. Right panels: movements are tagged by the identified type of movement produced. WF: wrist flexion, WE: wrist extension, FE: finger extension, UD: ulnar deviation, S: supination, WF+FF: wrist flexion and finger flexion, WF+FE: wrist flexion and finger extension, WF+UD: wrist flexion and ulnar deviation, WE+FE: wrist extension and finger extension, FE+UD: finger extension and ulnar deviation. Bottom panels in a) correspond to those of Fig. 5e-f.

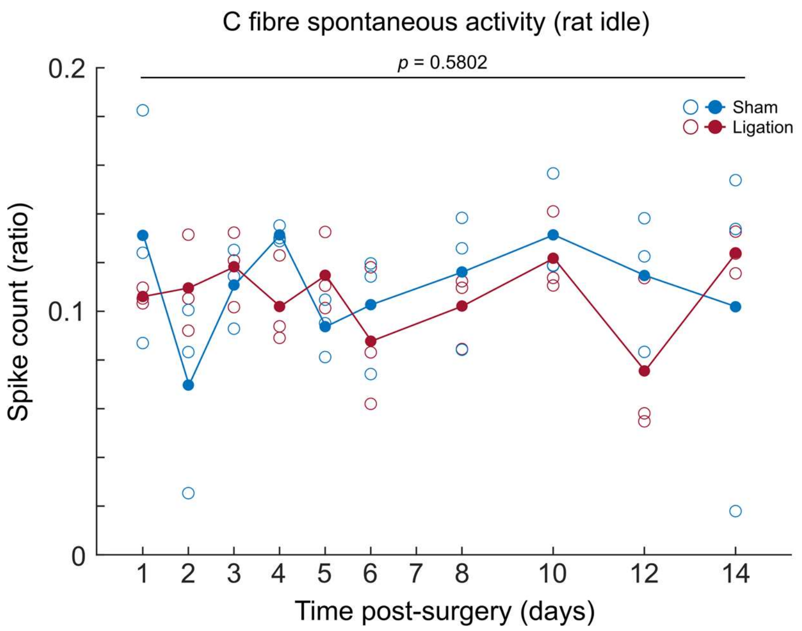

Supplementary Fig. 9 | C fibre activity (spike count as ratio to total spike count) in idle animals with or without partial nerve ligation over different intervals post-surgery. Statistical comparisons by two-way ANOVA (across all timepoints). Source data are provided as a Source Data file.
