# Supplementary material for: Ultraconformable cuff implants for long-term bidirectional interfacing of peripheral nerves at sub-nerve resolutions
Source: Nat Commun. 2024 Aug 30;15:7523. doi: 10.1038/s41467-024-51988-1 (PMC11364531; doi:10.1038/s41467-024-51988-1)
Supplement: Supplementary file 3 — Description of Additional Supplementary Files [file 41467_2024_51988_MOESM3_ESM.pdf]

Description of Additional Supplementary Files

Supplementary Movie 1| Video recordings of animals performing a dexterity task following ultraconformable device implantation. Videos presented for three different rats at Day 7 postimplantation. All three examples shown are scored as Grip (see Supplementary Fig. 3). Note implantation and task are performed over right front paw.

Supplementary Movie 2| Examples of other types of paw movements produced through stimulation with two microelectrodes simultaneously and different stimulation parameters. First (Finger extension + Wrist flexion) and second (Wrist extension + Wrist flexion) clips correspond to a one rat, while the third (Finger extension + Finger flexion) corresponds to a different rat. Microelectrodes delivering stimulation are depicted in red in implant diagram. The last portion of the video shows movements resulting from single pulses of stimulation, rather than 1 s pulse trains.
